# Socs3b regulates the development and function of innate immune cells in zebrafish

**DOI:** 10.3389/fimmu.2023.1119727

**Published:** 2023-03-08

**Authors:** Mohamed L. Sobah, Aimee C. Scott, Miranda Laird, Cassandra Koole, Clifford Liongue, Alister C. Ward

**Affiliations:** ^1^ School of Medicine, Deakin University, Geelong, VIC, Australia; ^2^ Institue for Mental and Physical Health and Clinical Translation (IMPACT), Deakin University, Geelong, VIC, Australia

**Keywords:** socs3, zebrafish, innate immunity, inflammation, cytokine signaling

## Abstract

**Introduction:**

Suppressor of cytokine signaling 3 (SOCS3) is a critical component of the negative feedback regulation that controls signaling by cytokines and other factors thereby ensuring that important processes such as hematopoiesis and inflammation occur at appropriate levels.

**Methods:**

To gain further insights into SOCS3 function, the zebrafish *socs3b* gene was investigated through analysis of a knockout line generated using CRISPR/Cas9-mediated genome editing.

**Results:**

Zebrafish *socs3b* knockout embryos displayed elevated numbers of neutrophils during primitive and definitive hematopoiesis but macrophage numbers were not altered. However, the absence of *socs3b* reduced neutrophil functionality but enhanced macrophage responses. Adult *socs3b* knockout zebrafish displayed reduced survival that correlated with an eye pathology involving extensive infiltration of neutrophils and macrophages along with immune cell dysregulation in other tissues.

**Discussion:**

These findings identify a conserved role for Socs3b in the regulation of neutrophil production and macrophage activation.

## 1 Introduction

The suppressor of cytokine signaling (SOCS) proteins regulate intracellular signaling induced by numerous extracellular regulators (1). Among the seven mammalian SOCS proteins, SOCS1-3 and the cytokine-inducible SH2-containing (CISH) protein are directly involved in the negative feedback control of cytokine receptor signaling, exerting a significant influence on processes such as hematopoiesis and innate immunity (1, 2). SOCS3 is highly expressed within the myeloid lineage, regulating both the differentiation and activation of neutrophils and macrophages (3, 4). Increased expression of SOCS3 has been observed in various models of chronic inflammation, corresponding with the degree of inflammation as well as the onset of various symptoms (5–7). However, decreased expression of SOCS3 is associated with the development of other inflammatory conditions, such as acute lung injury, hypersensitivity, and rheumatoid arthritis (8–10).

Global SOCS3 knockout mice exhibited embryonic lethality due to neutrophil-mediated inflammation, primarily within the placenta (11). This has been largely attributed to the role of SOCS3 in regulating signaling downstream of the granulocyte colony-stimulating factor receptor (G-CSFR) (12), with G-CSF stimulation of these mice resulting in uncontrolled inflammation and neutrophilic infiltration into various tissues, such as the liver, spleen and spine (4, 13). Hematopoietic specific deletion of SOCS3 also triggered a similar phenotype during late adulthood, with neutrophilia observed in both liver and lung, concurrent with elevated levels of IL-6 and G-CSF in these tissues (13).

The role of SOCS3 within the monocyte/macrophage lineage remains vague. In response to infections, activated macrophages displayed an upregulation of SOCS3 both *in vitro* and *in vivo* (14). Moreover, SOCS3-deficient macrophages showed increased expression of pro-inflammatory cytokines, such as IL-6, IL-1β and IL-12, as well as prolonged signaling through the JAK/STAT pathway in response to LPS (15). Furthermore, myeloid-specific deletion of SOCS3 exacerbated acute lung injury in response to LPS (7, 9). Lastly, the phagocytic abilities of macrophages were found to be upregulated following SOCS3 ablation, which has been attributed to enhanced IL-6 and IL-12 signaling (15, 16).

To better understand the *in vivo* function of SOCS3, this study generated a knockout mutant of the zebrafish *socs3b* gene (17). This mutant showed significantly elevated neutrophils throughout the life course although functional deficits were identified, while macrophages were more responsive to wounding. Adult *socs3b* mutant zebrafish developed a pathology of the eye, with infiltration of neutrophils into multiple tissues, suggesting a complex inflammatory disorder.

## 2 Methods

### 2.1 Zebrafish husbandry and line generation

Wildtype (WT), transgenic and mutant zebrafish were maintained in accordance with standard husbandry practices (18). To generate *socs3b* mutants, WT embryos were injected with ~1 pL 12.5 ng/μL guide RNA (gRNA)1 (5’-GUCCGAGCUGUCCCGCACC) and gRNA2 (5’-GGCGCACGCCGCCUUCAAAG) targeting *socs3b* and 100 ng/μL Cas9 mRNA (Sigma-Aldrich) at the 1 cell stage. Zebrafish were screened using PCR of genomic DNA extracted from fin clips using primers flanking the gRNA1 (5’-ACAGGGGGTGGGCAGAGC and 5’-GCTCACGGTGCTCCAGTAGAA) and gRNA2 (5’-GAGCGGGAAGGAGGCCAG and 5’-GCCGTTTTCACACTGAGCGT) target sites, followed by Sanger sequencing (Australian Genome Research Facility). Zebrafish carrying a suitable mutant allele were subsequently out-crossed with WT zebrafish for two generations. In order to minimize potential bias a mixed pool of embryos obtained from heterozygote mutant in-crosses were used, with genotyping performed post-analysis, as described (19). All experiments compared homozygous mutant zebrafish with WT siblings generated from the same cross and were performed in a blinded manner and repeated at least 3 times, with representative data shown. The mutant alleles were also crossed onto two transgenic lines Tg(*mpx*::GFP) (20) and Tg(*mpeg1.1*::GFP) (21).

### 2.2 Whole-mount *in situ* hybridization and Sudan black staining

Zebrafish embryos at relevant timepoints were fixed with 4% (*w/v*) paraformaldehyde (PFA) at 4°C prior to whole-mount *in situ* hybridization (WISH) using DIG-labelled anti-sense probes as previously described (22), with embryos younger than 22 hours post fertilization (hpf) dechorionated prior to fixing, or subjected to Sudan black staining (23). For probes that identified discrete cells (*spi1*, *lyz*, *lcp1*, *mpeg1.1*, *mpx*, *csf3r*, Sudan black), manual counting was performed across the embryos. For probes that showed staining in specific loci (*hbbe1.1*, *rag1*), the area was measured using ImageJ as the average from imaging both sides of the embryo (24). The region covering the majority of cells are shown in the respective figures.

### 2.3 Reverse-transcription polymerase chain reaction

Total RNA was extracted from zebrafish embryos and various adult tissues with either RNeasy Mini Kit (Qiagen) or Trisure (Bioline) according to the respective manufacturer’s protocol, depending on the nature of the sample. This was subjected to quantitative real-time reverse-transcriptase PCR (qRT^2^-PCR) with the primers listed in **Table 1**. Data were normalized relative to *actb* and fold-change calculated using the ΔCT method (31).

**Table 1:** Primers used for qRT^2^-PCR.

Gene	Accession Number	Primers	References
*actb*	NM131031	5’-TGGCATCACACCTTCTAC 5’-AGACCATCACCAGAGTCC	(25)
*ccr2*	XM_001344115	5’-TGGCAACGCAAAGGCTTTCAGTGA 5’-TCAGCTAGGGCTAGGTTGAAGAG	(26)
*cd4*	NM_001135096	5’-CTACCCAGAGAAAAGATTGAACG 5’-AGAAATCTGCTGATAGAGAGACG	This paper
*cd79*	NM_001326470.1	5’-GGAGTTATATGCCATTCGCTG 5’-ATTCTCTTCCTCCCTTTCTGTC	This paper
*cd8*	NM_001040049	5’-ACTCTTCTTCGGAGAGGTGAC 5’-ACAGGCTTCAGTGTTGTTTGAA	(27)
*cmyb*	AF191559	5’-GGTCCTCATGCCAAGTCAG 5’-CGGAGTTGGGCTGACTTTAG	This paper
*csf3b*	NM_001143754	5’-GTGTGCAGCGGATGCTCAT 5’-CTGCGAGGTCGTTCAGTAGGTT	This paper
*cxcr3.2*	NM_001007314	5’-CTCTGTTGGTAATGCTGTATTGC 5’-CACGATGACTAAGGAGATGA	(26)
*cxcr4b*	AY057094	5’-CCCATCACAAGCACCACAAG 5’-CGATAGCATCATTTTAGACAACAG	(26)
*cxcl8a*	XM_009306855	5’-CCAGCTGAACTGAGCTCCTC 5’-GGAGATCTGTCTGGACCCCT	(28)
*gata1b*	XM_021478544	5’-TTACTGCCAACCCGTTGATG 5’-GGAGAGTTTAGAGAGTGACCTGC	This paper
*hbaa1.1*	NM_131257	5’-GGGAGGTCTTGAGAGAGCC 5’-GCAATCAGCGAGAAGCCTGA	(29)
*ifng1*	NM_212864	5’-CTCAAAATGGTGCTACTCTGTG 5’-CAGCATTCCTTCAAGCAACAC	This paper
*ighm*	AF281479	5’-AAAGATTTGAGCGATTTTGTGC 5’-GCTAAACACATGAAGGTTGCTG	This paper
*ikzf1*	NM_130986	5’-AAGCGAAGTCACACTGAAGAAAG 5’-CAGATGTCCAGTGAGAGCGTC	This paper
*il1b*	NM_212844	5’-GGACTTCGCAGCACAAAATG 5’-GTTCACTTCACGCTCTTGGATG	(26)
*il21*	NM_001128574	5’-GAACTCAAGAAGATTCAGCAGG 5’-GTTCGGCTGTTGACCATTG	This paper
*il4*	NM_001170740	5’- CTGAATGGGAAAGGGGAAA 5’-CGAGAAACTCCTTCATTGTGC	This paper
*il6*	NM_001261449	5’-TCAGAGACGAGCAGTTTGAGAGAG 5’-GTTAGACATCTTTCCGTGCTG	This paper
*mpeg1.1*	NM_212737	5’-GTTACAGCACGGGTTCAA 5’-TGTTTTCAATGGCGTCAGC	This paper
*mpx*	NM_001351837	5’-CTGCGGGACCTTACTAATGATGG 5′-CCTGGATATGGTCCAAGGTGTC	(30)
*nkl11.3*	NM_001311793.1	5’-TGTGCTGCTCACTTGGAGATGC 5’-CATAGTCCAGGCAGTTGTTCC	This paper
*runx1*	NM_131603	5’-AGCTCGTCCCCATATCACCT 5’-TTGGTGCAGGGTAGGATTCG	(29)
*scl*	NM_213237	5’-GCTTTCCCTCTCCCGGCACG 5’-GTTCGTGAAAATCCGTCGCA	This paper
*tcra*	AF425590	5’-ACTGAAGTGAAGCCGAAT 5’-CGTTAGCTCATCCACGCT	This paper
*tgfb1b*	XM_687246	5’-AAATAGCAGGTTTGTCCCGC 5’-CACTTCCAGCCCAGGTCTT	(26)
*tnfa*	NM_212859	5’-GACTGAGGAACAAGTGCTTATGAG 5’-TGCCCAGTCTGTCTCCTTCTC	(26)
*tpor*	BN000861	5’-TACTTCCGAAAGGTCAAGAGGTC 5’-GACAGGTGTTCTTACAGGTTTTG	This paper

### 2.4 *Ex vivo* analyses

Adult zebrafish kidney cells were prepared in ice-cold phosphate buffered saline supplemented with 2 mM EDTA and 2% (*v/v*) fetal calf serum and passed through a 40 μm filter. For flow cytometry analysis, samples were analyzed on a FACSCanto II. Dead cells and doublets were removed by gating with lymphoid, erythroid, myeloid and precursor cells identified in a side scatter/forward scatter (SSC/FSC) plot and neutrophils and macrophages *via* GFP fluorescence using fish on the transgenic Tg(*mpx*::GFP) and Tg(*mpeg1.1*::GFP) backgrounds, respectively. Smears of kidney cells and adult blood were prepared using a cytospin and stained with Giemsa prior to differential counting and imaging using a Leica DME stereomicroscope and Olympus SC50 camera.

### 2.5 *In vivo* analyses

A wounding assay was utilized to identify relative neutrophil and macrophage migration using the transgenic lines and fluorescence microscopy (32). Embryos at 5 days post fertilization (dpf) were anaesthetized using 5 μg/mL benzocaine in system water. After the embryos stopped moving, an excision was made at the tip of the caudal fin with a number 21 scalpel. Embryos were then placed into 12 well plates containing 0.003% (w/v) PTU in system water and imaged at 2 hour intervals in the presence of 5 μg/mL benzocaine before being placed in fresh PTU solution to remove the benzocaine. Embryos were maintained on a heat block set at 28.5°C for the 24 hours of the assay and imaged on an Olympus MVX10 microscope with a DP74 camera using CellSens Dimension 1.6 software (Olympus) with UV excitation and a GFP filter for analysis. Cell numbers in the whole embryo prior to wounding and at the injury site across the time-course were manually determined. Cell areas were quantified with ImageJ using images obtained at the injury site during the peak of cell migration. The net rate of cell migration was calculated from the peak cell number divided by the corresponding time post-wounding, and the net rate of cell egress from the reduction from the peak until 24 hours post-wounding (hpw). In other experiments embryos were injected at the 1 cell stage with *csf3a* mRNA or at 3 dpf with lipopolysaccharide (LPS) as described (33).

### 2.6 Statistics

Statistical analyses were performed using GraphPad Prism version 8. Normality of data was tested using the D’Agostino-Pearson omnibus normality test and unequal variances using the F-test. For normally distributed data, the difference between two groups was analyzed for significance with a student *t*-test, with Welch’s correction if unequal variances were present. Where multiple *t*-tests were performed, the Bonferroni-Dunn method was used to correct for multiple comparisons. The difference between more than two groups was tested for significance using ordinary one-way ANOVA coupled with Tukey’s multiple comparison test. If data did not follow a normal distribution, the Mann-Whitney U test was performed. Survival and pathology incidence were visualized with a Kaplan-Meier graph, with the Gehan-Breslow-Wilcoxon test used to compare between different groups.

### 2.7 Sequence analysis

Protein sequences of human SOCS3 (NP_003946) as well as zebrafish Socs3a (NP_956244) and Socs3b (NP_998469) were obtained from GenBank and subjected to constraint-based multiple sequence analysis with COBALT (34).

## 3 Results

### 3.1 Generation of *socs3b* KO zebrafish

**Figure 1:**
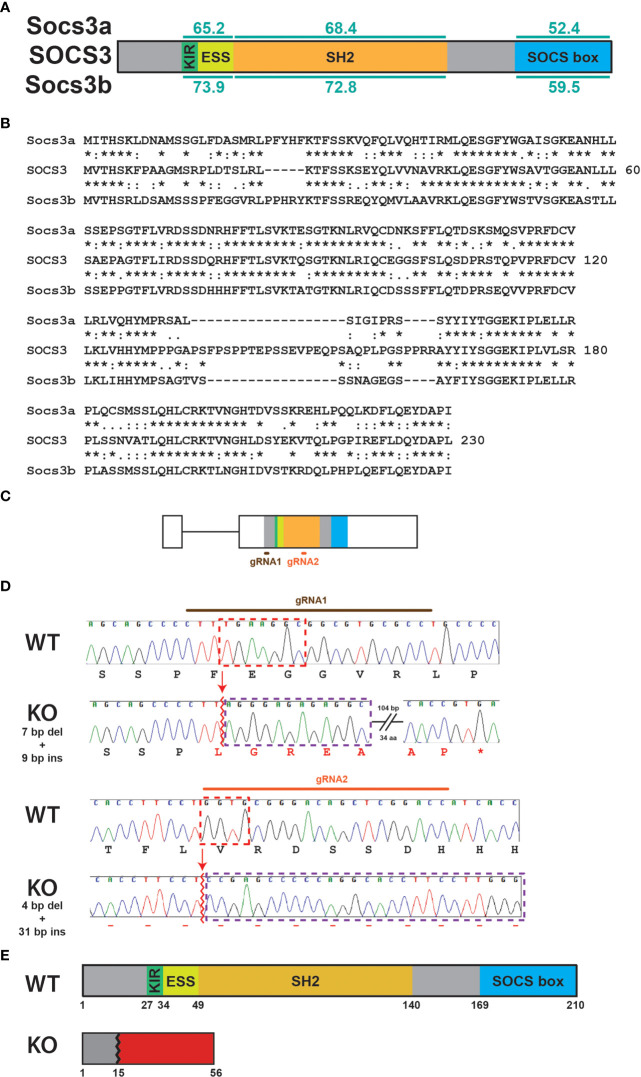
Generation of *socs3b* KO allele. Schematic of the SOCS3 protein displaying conserved functional domains (KIR: kinase inhibitory region, ESS: extended SH2 sub-domain, SH2: Src homology 2 domain, SOCS box) along with the relative conservation (% identity) of zebrafish Socs3a and Socs3b to the indicated domains **(A)**, as well as alignment of SOCS3 with Socs3a and Socs3b sequences **(B)**. The zebrafish *socs3b* gene structure, showing intron (thin line) and exons (boxes) and sequences targeted with gRNAs **(C)**. Sequence traces of representative homozygous wildtype (WT, *socs3b^wt/wt^*) and knockout (KO, *socs3b^mdu24/mdu24^*) fish around the sites targeted with gRNA1 (upper) or gRNA2 (lower), including nucleotides and encoded amino acids. Nucleotide sequences deleted are boxed red and those inserted boxed purple, with *de novo* protein sequences shown in red **(D)**. Schematic of Socs3b protein forms expressed in WT and KO fish **(E)**.

Both zebrafish Socs3a and Socs3b proteins share the same domain structure as human SOCS3 with very good overall conservation (**Figure 1A**), including many stretches of identical residues (**Figure 1B**). However, Socs3b shows higher conservation across the protein (**Figures 1A, B**) and so was chosen for further study. The *socs3b* gene also has the same exon structure as *SOCS3* (17), with exon 2 targeted using two guide RNAs (gRNAs) directed at sequences encoding the N-terminus and SH2 domain (**Figure 1C**). These were injected along with Cas9 mRNA into embryos, which were raised to adulthood and out-crossed with wildtype (WT) fish. Screening of the resultant F1 fish identified a single mutant allele that impacted the *socs3b* reading frame. The carrier of this allele was again out-crossed before in-crossing to produce homozygous F3 mutant fish. Sequence analysis revealed that this allele, designated *mdu24*, possessed a complex insertion/deletion at both gRNA sites (**Figure 1D**). The first caused a frameshift resulting in 41 *de novo* residues after proline at position 15 followed by a stop codon, with the second occurring after this point and so not impacting the protein encoded, which represented a severely truncated knockout (KO) version of the Socs3b protein, lacking the SH2 and SOCS box domains (**Figure 1E**).

### 3.2 *socs3b* regulates the number of primitive and early definitive neutrophils

**Figure 2:**
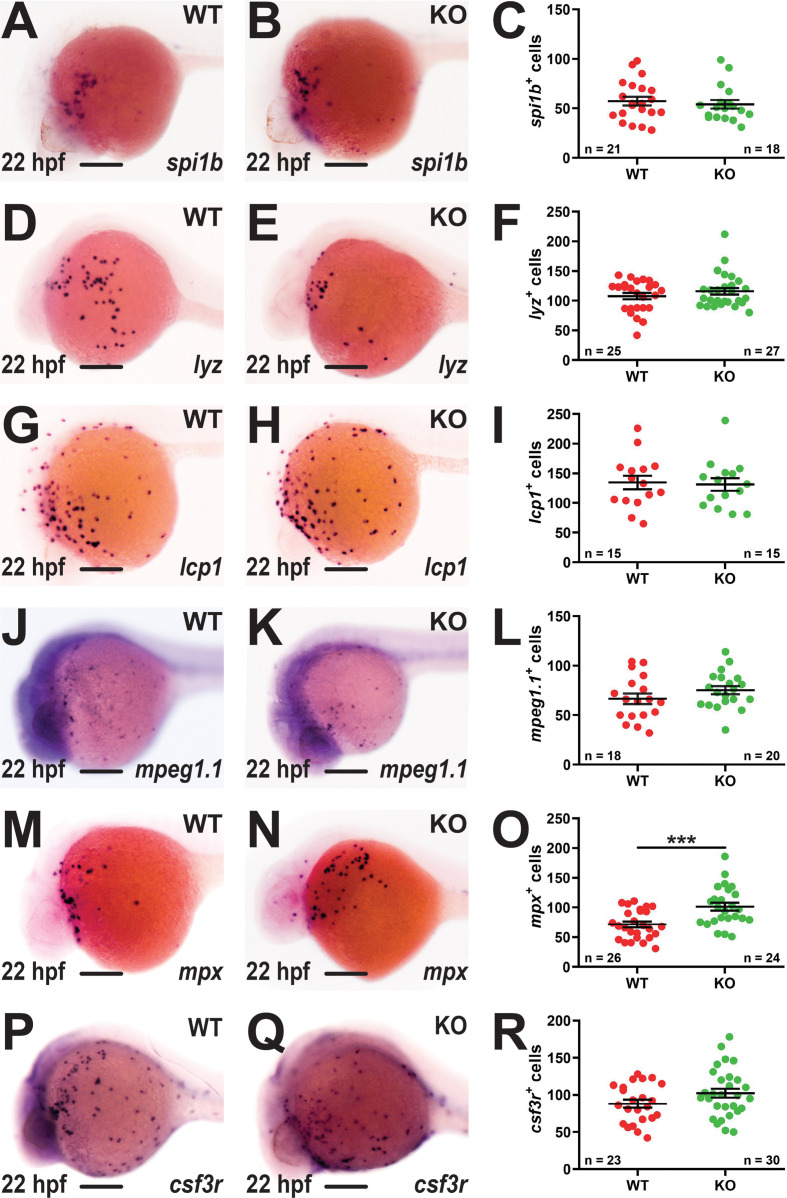
Effect of *socs3b* ablation on primitive hematopoiesis. Representative images of wildtype (WT, *socs3b^wt/wt^*) and knockout (KO, *socs3b^mdu24/mdu24^*) embryos subjected to WISH using *spi1b* **(A, B)**, *lyz* **(D, E)**, *lcp1* **(G, H)**, *mpeg1.1* **(J, K)**, *mpx* **(M, N)** and *csf3r* **(P, Q)** at 22 hpf, showing the major areas of staining in each case, with scale bars representing 200 μm. Quantitation of *spi1b^+^* **(C)**, *lyz^+^* **(F)**, *lcp1^+^* **(I)**, *mpeg1.1^+^* **(L)**, *mpx^+^* **(O)** and *csf3r^+^* **(R)** cells showing numbers in individual embryos along with mean and standard error of the mean (SEM), with statistical significance indicated. Student *t*-tests were used to measure the significance between WT and KO samples with Welch’s correction performed if required (*** *p* < 0.001).

The potential effects of *socs3b* ablation on primitive hematopoiesis were assessed using WISH with lineage-specific markers at 22 hpf. No difference was observed between WT and KO embryos with *spi1b*, a marker of early myeloid progenitors (25) (**Figures 2A–C**), or the pan-leucocyte markers *lyz* (35) (**Figures 2D–F**) and *lcp1* (36) (**Figures 2G–I**). Similarly, there was no difference between WT and KO embryos with the macrophage marker *mpeg1.1* (37) (**Figures 2J–L**). However, the neutrophil population, identified using *mpx* (38), was significantly increased in KO embryos (**Figures 2M–O**). The number of cells expressing the alternate neutrophil marker *csf3r* (33) were elevated but not to a statistically significant level (**Figures 2P–R**).

**Figure 3:**
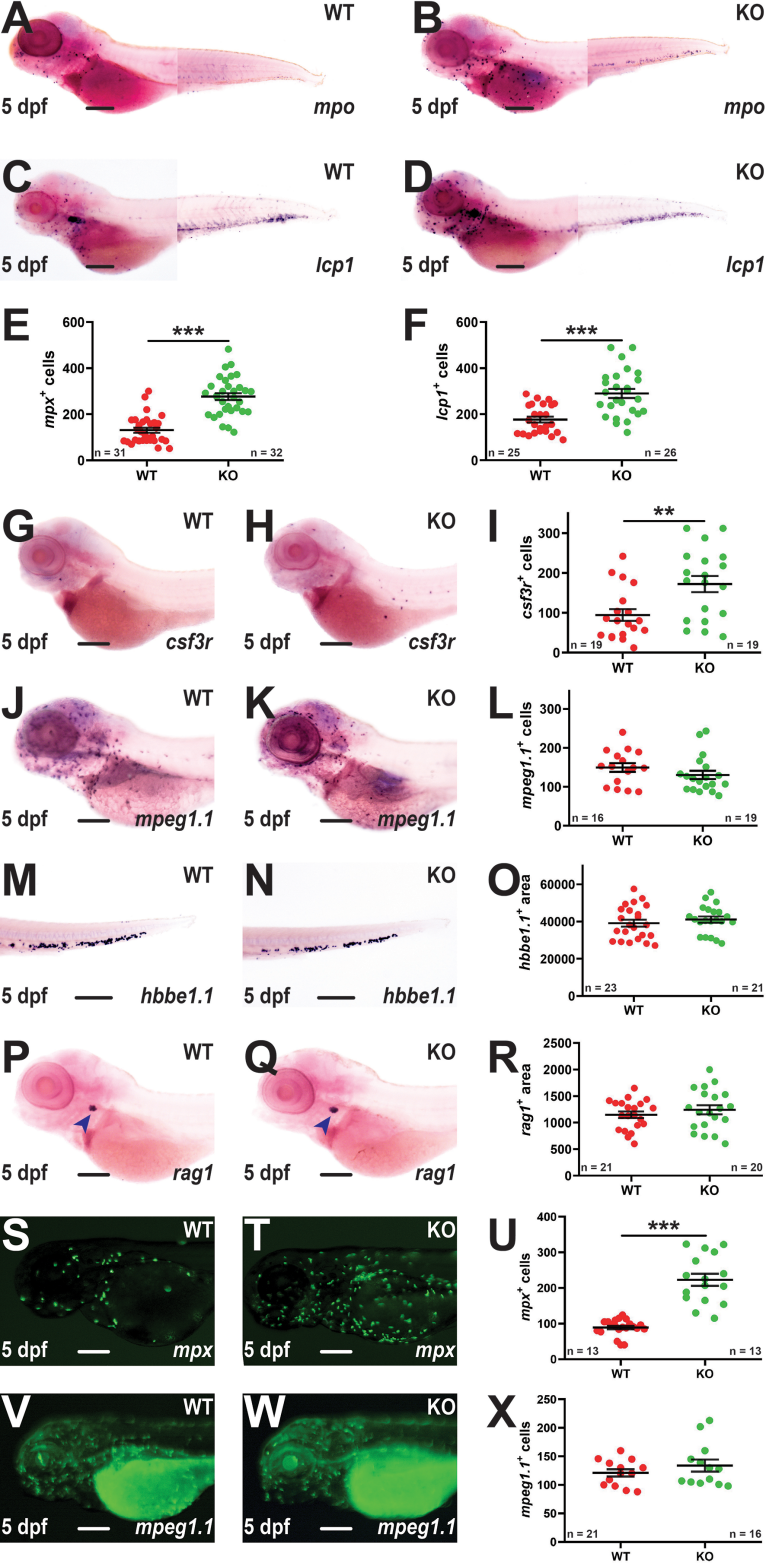
Effect of *socs3b* ablation on early definitive hematopoiesis. Representative images of wildtype (WT, *socs3b^wt/wt^*) and knockout (KO, *socs3b^mdu24/mdu24^*) embryos subjected to WISH using *mpx* **(A, B)**, *lcp1* **(C, D)**, *csf3r* **(G, H)**, *mpeg1.1* **(J, K)**, *hbbe1.1* **(M, N)** and *rag1* **(P, Q)** at 5 dpf, showing the major areas of staining in each case, with scale bars representing 200 μm. Fluorescent images of WT and KO embryos on either the Tg(*mpx*::GFP) **(S, T)** or Tg(*mpeg1.1*::GFP) **(V, W)** transgenic backgrounds, with scale bars representing 200 μm. Quantitation of *mpx^+^* **(E)**, *lcp1^+^* **(F)**, *csf3r^+^* **(I)**, *mpeg1.1^+^* **(L)** and GFP^+^ cells in Tg(*mpx*::GFP) **(U)** and Tg(*mpeg1.1*::GFP) **(X)** embryos, and area of staining for *hbbe1.1* **(O)** and *rag1* **(R)** showing values for individual embryos along with mean and SEM, with statistical significance indicated. Student *t*-tests were used to measure the significance between WT and KO with Welch’s correction performed if required (*** *p* < 0.001, ** *p* < 0.01).

Early definitive hematopoiesis was assessed using WISH at 5 dpf. A significant increase in *mpx^+^* cells was again observed in KO compared to WT embryos (**Figures 3A, B, E**). There was also a significant elevation in cells expressing *lcp1* (**Figures 3C, D, F**) or *csf3r* (**Figures 3G–I**), but not *mpeg1.1* (**Figures 3J–L**). Analysis of mature erythrocytes using *hbbe1.1* (39) (**Figures 3M–O**) and T lymphocytes using *rag1* (40) (**Figures 3P–R**) revealed no differences between WT and KO embryos. To further investigate the potential impact of *socs3b* on innate immune cells fish carrying the KO allele were separately out-crossed with two transgenic lines Tg(*mpx*::GFP) (20) and Tg(*mpeg1.1*::GFP) (21) to facilitate visualization of neutrophils and macrophages, respectively. This confirmed a significant elevation in the number of *mpx^+^* neutrophils (**Figures 3S–U**) but not *mpeg1.1^+^* macrophages (**Figures 3V–X**) in KO compared to WT embryos.

### 3.3 *socs3b* regulates the functionality of neutrophils and macrophages

**Figure 4:**
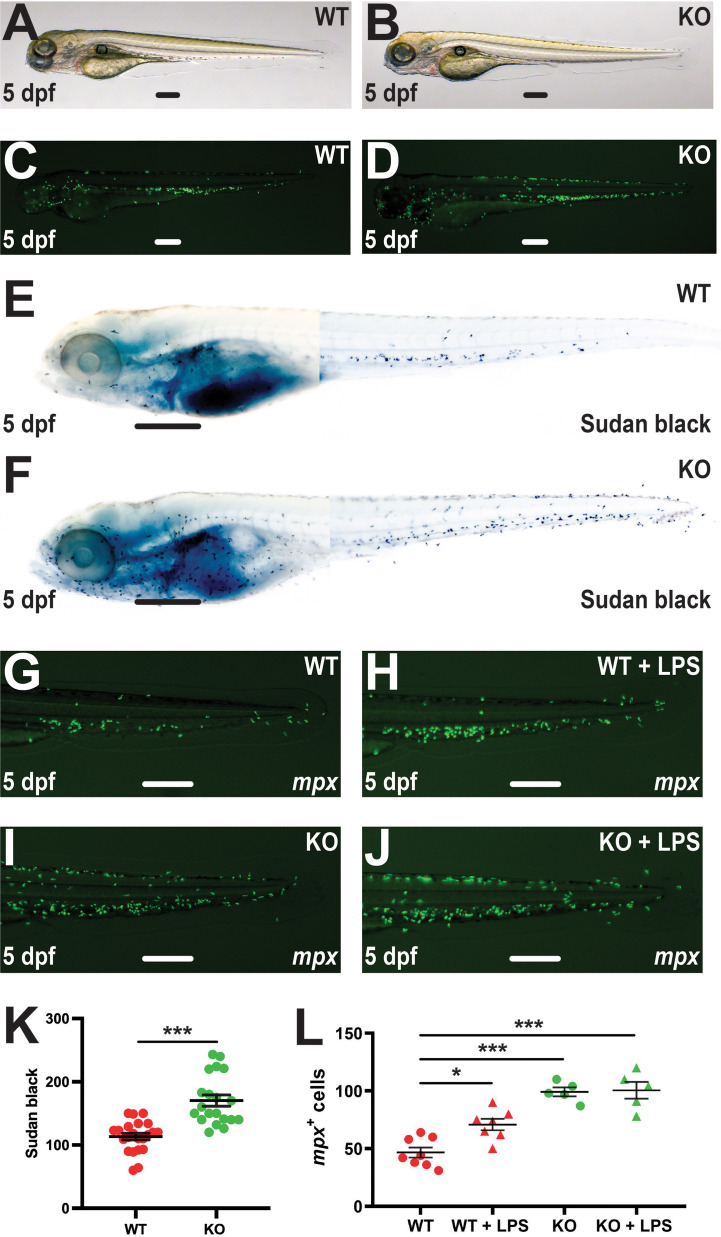
Effect of *socs3b* ablation on neutrophil maturation and functionality. Wildtype (WT, *socs3b^wt/wt^*) and knockout (KO, *socs3b^mdu24/mdu24^*) embryos on the Tg(*mpx*::GFP) transgenic background at 5 dpf imaged by light **(A, B)** and fluorescence **(C, D)** microscopy or subjected to Sudan black staining **(E, F)**, showing representative images in each case. Embryos were also subjected to microinjection with LPS at 5 dpf and imaged by fluorescence microscopy 8 hours later **(G–J)**, showing representative images. Scale bars represent 100 μm. Quantitation of Sudan black^+^ **(K)** or *mpx^+^* cells **(L)** with mean and SEM shown, with significance tested using one-way ANOVA (***p < 0.001, *p < 0.05, n = 8-12).

Further analysis of the Tg(*mpx*::GFP) embryos by light microscopy failed to detect any differences between WT and KO genotypes (**Figures 4A, B**), while fluorescence microscopy revealed similar distribution of neutrophils across the embryo (**Figures 4C, D**). To assess neutrophil maturation, 5 dpf embryos were stained with Sudan black as a marker of differentiation (23) (**Figures 4E, F**). The number of stained cells was again significantly higher in KO compared to WT embryos with no difference in staining intensity observed (**Figure 4K**), suggesting the increased neutrophils were mature. To further investigate neutrophil responses, lipopolysaccharide (LPS) was also injected into WT and KO embryos on the Tg(*mpx*::GFP) transgenic background to simulate bacterial infection (33). In WT embryos LPS induction was able to significantly increase the *mpx^+^* neutrophil population, but this increase was not observed in KO embryos (**Figures 4G–J, L**).

**Figure 5:**
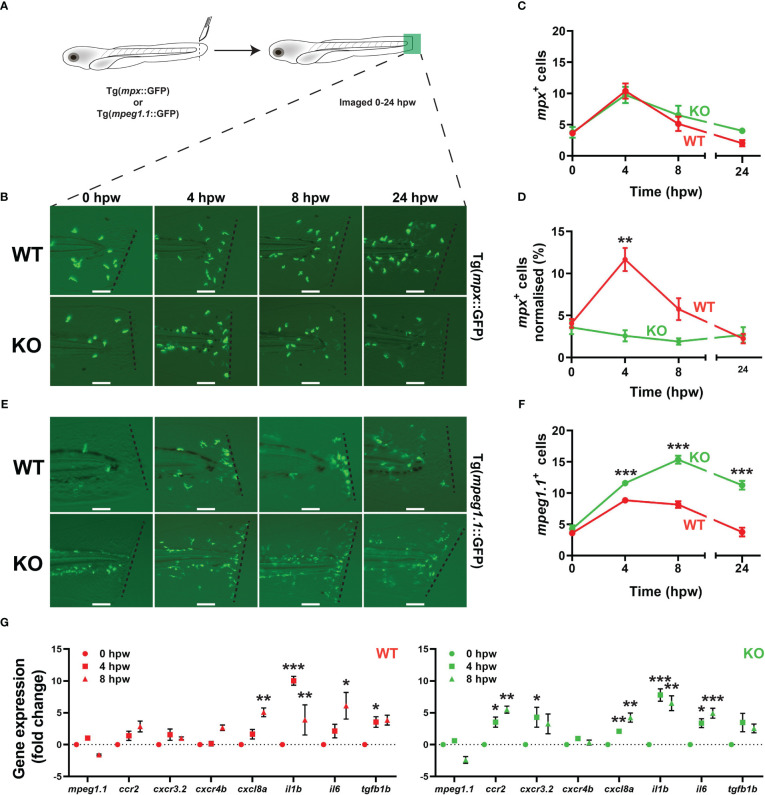
Effect of *socs3b* ablation on the innate immune cell response to injury. Wildtype (WT, *socs3b^wt/wt^*) and knockout (KO, *socs3b^mdu24/mdu24^*) embryos on either the Tg(*mpx*::GFP) or Tg(*mpeg*1.1::GFP) transgenic background were subjected to a tail wounding assay at 5 dpf and imaged under fluorescence from 0-24 hours post wounding (hpw) **(A)**. Representative images of *mpx* ^+^ neutrophil **(B)** and *mpeg1.1* ^+^ macrophage **(E)** cell migration to the injury site (dotted line) at the indicated times, with scale bars representing 100 mm. Quantitation of all migrating *mpx* ^+^ **(C)** and normalized to total *mpx* ^+^ cells **(D)** as well as all *mpeg1.1* ^+^ cells **(F)**. Gene expression analysis of the indicated genes in WT and KO embryos subjected to wounding presented as fold-change (log_2_) relative to 0 hpw **(G)**. Panels **(C, D, F** and **G)** show means and SEM, with statistical significance indicated using unpaired *t* tests **(C, D** and **F)** or one-way ANOVA **(G)** (*** *p* < 0.001, ** *p* < 0.01, * *p* < 0.05, n = 8-12).

Embryos derived from both transgenic lines were also subjected to a tail wounding assay at 5 dpf (**Figure 5A**). No difference was observed in the neutrophil response to injury between WT and KO embryos, with similar numbers and kinetics of *mpx* ^+^ cells observed (**Figures 5B, C**). However, when this data was normalized with respect to total *mpx^+^* cells, a significantly lower neutrophil response to wounding in KO embryos compared to WT was apparent (**Figure 5D**). In contrast, a significantly larger and more sustained response was seen for *mpeg1.1^+^* macrophages in KO compared to WT embryos (**Figures 5E, F**). There was no difference between WT and KO embryos in relative size (**Supplementary Figures 1A, B**) or egress rate (**Supplementary Figures 1C, D**) for *mpx^+^* or *mpeg1.1^+^* cells during wounding, but there was a significant increase in the rate of migration for *mpeg1.1^+^* (**Supplementary Figure 1F**) but not *mpx^+^* (**Supplementary Figure 1E**) cells in KO embryos. Gene expression analysis revealed a significant increase in the inflammatory markers *cxc8la*, *il1b*, *il6* and *tgfb1b* (41, 42) in both WT and KO embryos after wounding but two markers of inflammatory macrophages *ccr2* and *cxcr3.2* (42) were significantly upregulated only in KO embryos (**Figure 5G**). Comparing gene expression between genotypes at the various timepoints revealed elevation of *mpeg1.1*, *ccr2* and *il6* basally and all genes by 8 hpw in KO compared to WT fish (**Supplementary Figure 2**).

### 3.4 *socs3b* impacts steady-state adult myeloid populations in zebrafish

**Figure 6:**
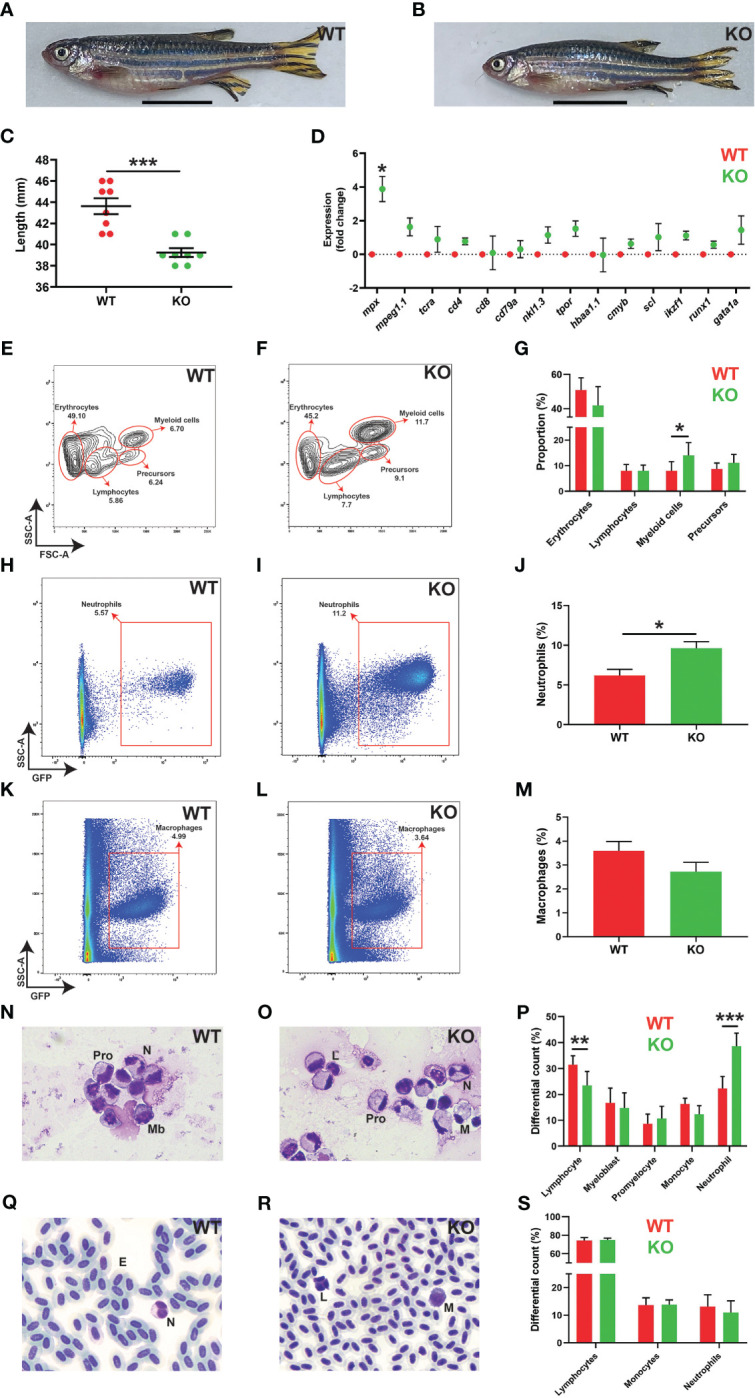
Effects of *socs3b* ablation on adult development and hematopoiesis. Representative images of wildtype (WT, *socs3b^wt/wt^*) and knockout (KO, *socs3b^mdu24/mdu24^*) zebrafish at 5 months post fertilization (mpf) **(A, B)**, with scale bars representing 1 cm, along with quantitation of their size showing results for individual fish along with mean and SEM, with significance tested using a parametric *t*-tests (n = 8) **(C)**. Gene expression analysis of the indicated genes in WT and KO adult kidney presented as fold change (log_2_) relative to WT, showing mean and SEM. Normalized Cq values obtained from WT and KO kidney marrow was used to test for significance using parametric *t*-tests (n = 6) **(D)**. Flow cytometry analysis of WT and KO kidney for erythrocyte, lymphocyte, myeloid cell and precursor **(E, F)** populations, with analysis of *mpx+* neutrophils **(H, I)** and *mpeg1.1+* macrophages **(K, L)** as well as quantitation of relevant populations **(G, J, M)** showing mean and SEM with significance tested using *t*-tests with Welch’s correction (n = 10) (*** *p* < 0.001, ** *p* < 0.01, * *p* < 0.05). Histological analysis of adult kidney **(N, O)** and blood **(Q, R)**, showing representative images, as well as quantitation of both **(P, S)**, showing mean and SEM, a parametric *t*-tests (n = 6 and n = 8, respectively) **(F)**. Mb, myeloblast; Pro, promyelocyte; M, monocyte; L, lymphocyte; N, neutrophil; E, erythrocyte.

Adult KO zebrafish had a major sex bias, with females drastically underrepresented, meaning it was only feasible to characterize adult male KO zebrafish. These were significantly smaller than male WT zebrafish at 5 months post fertilization (mpf), but otherwise anatomically normal (**Figures 6A–C**). Gene expression analysis of the kidney, which is the equivalent of mammalian bone marrow with respect to adult hematopoiesis (43), revealed a significant and specific increase in the neutrophil marker *mpx* in *socs3b* KO compared to WT fish, but not markers of macrophages (*mpeg1.1*) (21), T cells (*tcra*, *cd4*, *cd8*) (44), B cells (*cd79a*) (45), NK cells (*nkl1.3*) (46), thrombocytes (*tpor*) (47), RBC (*hbaa1.1*) (47), or precursors (*cmyb*, *scl*, *ikzf1*, *runx1*, *gata1a*) (48–50) (**Figure 6D**). Forward and side scatter analysis of kidney cells also identified a significantly larger myeloid cell compartment in KO zebrafish (**Figures 6E–G**). Moreover, analysis of fish on the Tg(*mpx::*GFP) and Tg(*mpeg1.1::*GFP) backgrounds revealed a significant increase in kidney neutrophils (**Figures 6H–J**) – but not macrophages (**Figures 6K–M**) – in KO compared to WT fish. Analysis of kidney smears confirmed a specific increase in mature neutrophils in KO fish that were morphologically indistinguishable from WT (**Figures 6N–P**), but this increase was not reflected in blood smears in which neutrophil numbers were not significantly different between genotypes (**Figures 6Q–S**).

### 3.5 *socs3b* KO zebrafish suffer from chronic inflammation during adulthood

**Figure 7:**
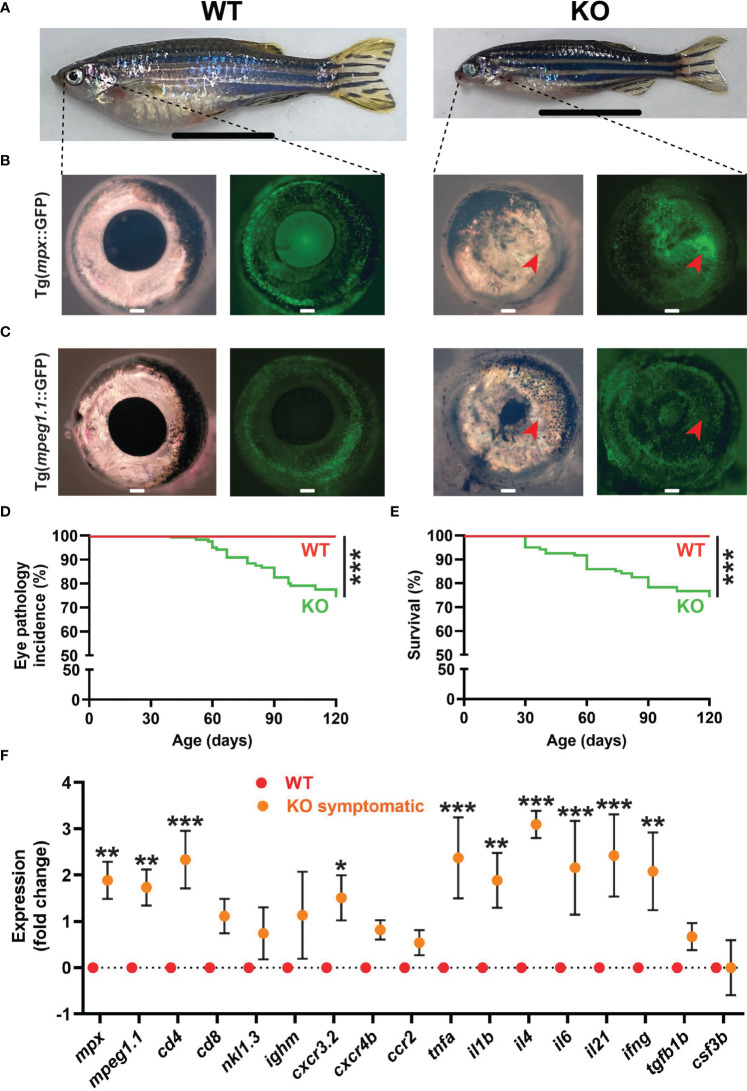
Eye pathology in *socs3b* KO zebrafish. Representative images of wildtype (WT, *socs3b^wt/wt^*) and symptomatic knockout (KO, *socs3b^mdu24/mdu24^*) zebrafish on either the Tg(*mpx*::GFP) **(A, B)** or Tg(*mpeg1.1*::GFP) **(C)** background, with close-up of right eye from respective zebrafish **(B, C)** under light (left panels) and fluorescence (right panels) microscopy, with scale bar representing 1 cm. Incidence of eye pathology **(D)** and overall survival **(E)** in WT and KO zebrafish (n = 25), with significance determined using a log-rank test. Gene expression analysis of WT and symptomatic KO adult eye showing mean and SEM (n = 6) **(F)**. Normalized Cq values obtained from WT and KO eyes was used to test for significance using parametric *t*-tests (*** *p* < 0.001, ** *p* < 0.01, * *p* < 0.05).

A significant proportion of KO zebrafish developed an eye pathology, with these fish eventually becoming emaciated (**Figure 7A**). Affected individuals displayed significant tissue growth on the surface of the eye, in most cases covering the pupil and resulting in the development of so-called ‘bug eye’ (51) (**Figures 7A–C**). This tissue contained both neutrophils and macrophages as revealed in Tg(*mpx::*GFP) (**Figure 7B**) and Tg(*mpeg1.1::*GFP) (**Figure 7C**) lines, respectively. This eye pathology was first evident at around 1 mpf and reached almost 30% by 4 mpf (**Figure 7D**), and strongly correlated with early mortality in these fish (**Figure 7E**). Gene expression analysis of whole eyes confirmed an upregulation of neutrophil (*mpx*) (10) and macrophage (*mpeg1.1*) (37) markers in symptomatic KO fish compared to WT fish, as well as one T cell marker (*cd4*) (52), although the other T cell marker (*cd8*) and those for B cells (*ighm*) (53) and NK cells (*nkl.3*) (46) were not significantly altered (**Figure 7F**). Several inflammatory macrophage markers (*cxcr3.2*, *tnfa*, *il1b*) (42) were upregulated, along with inflammatory cytokines *il4*, *il6*, *il21* (54) and *ifng* (55), but not *cxcr4b*, *ccr2*, *tgfb1b* or *csf3b* (**Figure 7F**). The gene expression profiles of various tissues extracted from normal WT and both symptomatic and asymptomatic KO adults were also assessed (**Supplementary Figure 3**). The neutrophil marker *mpx* was found to be significantly upregulated in the spleen and liver of symptomatic KO compared to WT, and compared to asymptomatic KO for liver, but was down-regulated in muscle (**Supplementary Figures 3A-C**). No significant change was observed in macrophage marker *mpeg1.1* in any of these tissues (**Supplementary Figures 3A–D**). The lymphocyte marker *cd8* was also significantly upregulated in both the liver and spleen of symptomatic and asymptomatic KO compared to WT, with *cd4* upregulated in asymptomatic KO only for these tissues (**Supplementary Figures 3A, B**).

## 4 Discussion

SOCS3 is strongly implicated in the regulation of innate immune cell production and function (56, 57), with its disruption associated with a range of inflammatory conditions (1). Zebrafish possess two paralogous genes, *socs3a* and *socs3b*, the encoded proteins of which show conservation of functional domains (17) and activation downstream of Stat3 (58). To provide further insight into the role of SOCS3 in innate immunity, CRISPR/Cas9-mediated genome editing was used to generate a *socs3b* KO mutant that lacked all functional domains. Fish harboring this mutation were viable into adulthood, in contrast to global *Socs3* knockout mice that suffered from embryonic lethality (11, 59), enabling analysis across the full life-course. These *socs3b* KO fish displayed enhanced neutrophil production, altered macrophage function, and developed an inflammatory phenotype during adulthood associated with a striking eye pathology and extensive neutrophil infiltration.

Zebrafish *socs3b* mutants showed a significant enhancement in the number of differentiated neutrophils during both the primitive and early definitive waves of hematopoiesis in embryos and extending to steady-state levels across multiple adult tissues. Morpholino-mediated knockdown of *socs3b* also elicited an increase in embryonic neutrophil numbers (data not shown) corroborating the results from the mutant allele. This is consistent with the neutrophilia observed in *Socs3* knockout mice, which included infiltration into multiple organs (4). Moreover, elevated *socs3b* mRNA levels observed in zebrafish Tet mutants correlated with defective neutrophil maturation (60) identifying Socs3b as a crucial regulator of neutrophil production. Signaling by G-CSFR plays a key role in this process (33), with *Socs3* knockout mice being hypersensitive to G-CSF stimulation (4, 13), suggesting unrestrained G-CSFR signaling as the underlying cause of the neutrophilia in these animals. Zebrafish G-CSFR has a conserved role in neutrophil production and function (33, 61, 62) and possesses SOCS3 binding sites in its intracellular domain like its mammalian homologues (33), with enforced G-CSF expression leading to strong *socs3b* induction (**Supplementary Figure 4A**). Together this indicates that dysregulated G-CSFR signaling likely also contributes to the increased neutrophils in zebrafish *socs3b* mutants.

Macrophage numbers were not altered in *socs3b* KO mutants, again consistent with mouse Socs3 knockouts (4, 13). However, embryonic macrophages were more responsive to injury in *socs3b* KO mutants, with increased number and sustained retention observed at the site of injury. This occurred concurrently with upregulation of chemokine receptors *cxcr3.2* and *ccr2*, both of which are synonymous with macrophage recruitment to injury where they play crucial roles in tissue repair (63–65). A significant increase in CD4+ and CD8+ T cells was also observed in the spleen and liver of *socs3b* mutants. This also has some precedence in mice, where SOCS3 has been demonstrated to regulate CD8+ T cell proliferation mediated by IL-6 and IL-27 (66). In contrast, neutrophil functionality was reduced in *socs3b* KO fish.

The zebrafish *socs3b* mutants developed an eye pathology that was associated with increased infiltration of neutrophils, macrophages and T-lymphocytes, as well as elevated expression of the genes encoding the cytokines IL-1β, TNF-α and IFN-γ. The eye pathology was reminiscent of autoimmune uveitis, showing similar characteristics including upregulation of TNF-α (67). Interestingly, T cell specific *Socs3* deletion protected mice from both chronic and acute experimental autoimmune uveitis (EAU) through an increase in regulatory T cells that produced higher levels of IL-10 (68). In contrast, myeloid specific *Socs3* deletion exacerbated the development of inflammation-mediated retinal degradation in EUA, with increased neutrophil infiltration and enhanced levels of IL-1β, TNF-α and IFN-γ (69). These mice also exhibited worsened experimental autoimmune encephalomyelitis (EAE), with infiltration of neutrophils, B-cells and Th1 cells into the brain, along with demyelination and increased levels of IFN-γ, IL-6 and IL-17, ultimately resulting in earlier lethality (15). Neutrophils in these mice showed heightened STAT3 activation in response to G-CSF, resulting in increased production of reactive oxygen species (ROS), with ablation of G-CSF ameliorating the severity of EAE suggesting this cytokine was responsible for the severe inflammation (70). Collectively, this suggests a role for excessive G-CSFR signaling in the neutrophil lineage in the eye pathology. Increased expression of *socs3b* has been associated with optic nerve pathology (71). It was also upregulated following optic nerve injury, but knockdown of *socs3b* had no effect on axon regeneration (30), suggesting a role outside the optic nerve. However, SOCS3 is also expressed in photoreceptor cells and plays a protective role in these cells during inflammation by suppressing STAT3 activation (72), indicating a more complex etiology is likely.

Collectively our observations are consistent with chronic systemic inflammation in the KO fish. Whether this is due to the increased number of neutrophils, their altered functionality, the enhanced responsiveness of macrophages or a combination of these remains to be determined, but could be explored by ablation of specific cell populations such as using NTR/metronidazole. The neutrophil dysregulation is particularly intriguing, since their maturation appears normal but their responsiveness is blunted. One key aspect may relate to mobilization/migration with decreased neutrophil migration to injury in embryos, but also normal circulating neutrophil numbers despite increased stores in the kidney. However, other critical neutrophil functions – such as phagocytosis, NETosis and ROS production – may also be impacted, any of which could contribute to the phenotypes observed. Further close examination of the eye pathology and the response to injury in adults would likely provide useful insights.

Multiple SOCS proteins have been implicated in the development of autoimmune and inflammatory diseases. Dysregulated expression of SOCS3 has been frequently observed in various forms of inflammatory disorders, such as rheumatoid arthritis and Crohn’s disease (73, 74), with SOCS1 haploinsufficiency shown to predispose to early onset autoimmune disease (75). CISH knockout mice showed increased susceptibility to experimental allergic asthma (76) and EAE (77), while SOCS5 was preferentially expressed in the retina and significantly upregulated during the development and resolution EAU (78). As a result therapeutic strategies targeting SOCS proteins have been developed to treat relevant diseases. For example, mimetic SOCS1 peptides were able to reduce intraocular inflammation and EAU development in mice by suppressing the activities of IFN-γ and TNF-α and thereby preventing ocular damage (79, 80). Cell-penetrating SOCS3 forms have also been shown to be efficacious in inhibiting inflammation, with inhibition of IFN-γ and TNF-α activity again observed (81, 82). Our zebrafish model is ideally set up to further explore potential therapeutic agents for SOCS3, which could be readily extended to other SOCS proteins. Further studies could also explore the role of Socs3a, with evidence that it may compensate following the loss of Socs3b and/or act in parallel (**Supplementary Figures 4B, C**).

## Data availability statement

The original contributions presented in the study are included in the article/**Supplementary Material**. Further inquiries can be directed to the corresponding author.

## Ethics statement

The animal study was reviewed and approved by Deakin Univesity Animal Ethics Committee.

## Author contributions

AW conceived the project. CK performed preliminary characterization using morpholinos. ML generated the zebrafish mutant, which was characterized by MS and AS. AW and CL co-supervised MS, AS, ML and CK and contributed to on-going experimental design. All authors contributed to the article and approved the submitted version.

## References

[1] Sobah ML Liongue C Ward AC. SOCS proteins in immunity, inflammatory diseases, and immune-related cancer. Front Med (2021) 8:727987. doi: 10.3389/fmed.2021.727987 PMC8481645 34604264

[2] Trengove MC Ward AC. SOCS proteins in development and disease. Am J Clin Exp Immunol (2013) 2:1–29. 23885323 PMC3714205

[3] Yu H Liu Y McFarland BC Deshane JS Hurst DR Ponnazhagan S. SOCS3 deficiency in myeloid cells promotes tumor development: involvement of STAT3 activation and myeloid-derived suppressor cells. Cancer Immunol Res (2015) 3:727–40. doi: 10.1158/2326-6066.CIR-15-0004 PMC4570503 25649351

[4] Croker BA Metcalf D Robb L Wei W Mifsud S DiRago L. SOCS3 is a critical physiological negative regulator of G-CSF signaling and emergency granulopoiesis. Immunity (2004) 20:153–65. doi: 10.1016/S1074-7613(04)00022-6 14975238

[5] Shaw EJ Smith EE Whittingham-Dowd J Hodges MD Else KJ Rigby RJ. Intestinal epithelial suppressor of cytokine signaling 3 (SOCS3) impacts on mucosal homeostasis in a model of chronic inflammation. Immune Inflamm Dis (2017) 5:336–45. doi: 10.1002/iid3.171 PMC5569373 28508554

[6] Draijer C Speth JM Penke LRK Zaslona Z Bazzill JD Lugogo N. Resident alveolar macrophage-derived vesicular SOCS3 dampens allergic airway inflammation. FASEB J (2020) 34:4718–31. doi: 10.1096/fj.201903089R PMC7433762 32030817

[7] Jiang Z Chen Z Li L Zhou W Zhu L. Lack of SOCS3 increases LPS-induced murine acute lung injury through modulation of Ly6C(+) macrophages. Respir Res (2017) 18:217. doi: 10.1186/s12931-017-0707-6 29284516 PMC5747159

[8] Boosani CS Agrawal DK. Methylation and microRNA-mediated epigenetic regulation of SOCS3. Mol Biol Rep (2015) 42:853–72. doi: 10.1007/s11033-015-3860-3 PMC5542822 25682267

[9] Yan C Ward PA Wang X Gao H. Myeloid depletion of SOCS3 enhances LPS-induced acute lung injury through CCAAT/enhancer binding protein δ pathway. FASEB J (2013) 27:2967–76. doi: 10.1096/fj.12-225797 PMC3714578 23585399

[10] Bennett CM Kanki JP Rhodes J Liu TX Paw BH Kieran MW. Myelopoiesis in the zebrafish, Danio rerio. Blood (2001) 98:643–51. doi: 10.1182/blood.V98.3.643 11468162

[11] Takahashi Y Carpino N Cross JC Torres M Parganas E Ihle JN. SOCS3: an essential regulator of LIF receptor signaling in trophoblast giant cell differentiation. EMBO J (2003) 22:372–84. doi: 10.1093/emboj/cdg057 PMC140741 12554639

[12] White CA Nicola NA. SOCS3: An essential physiological inhibitor of signaling by interleukin-6 and G-CSF family cytokines. JAK-STAT (2013) 2:e25045. doi: 10.4161/jkst.25045 24416642 PMC3876435

[13] Kimura A Kinjyo I Matsumura Y Mori H Mashima R Harada M. SOCS3 is a physiological negative regulator for granulopoiesis and granulocyte colony-stimulating factor receptor signaling. J Biol Chem (2004) 279:6905–10. doi: 10.1074/jbc.C300496200 14699146

[14] Arnold CE Whyte CS Gordon P Barker RN Rees AJ Wilson HM. A critical role for suppressor of cytokine signalling 3 in promoting M1 macrophage activation and function *in vitro* and *in vivo* . Immunology (2014) 141:96–110. doi: 10.1111/imm.12173 24088176 PMC3893853

[15] Qin H Holdbrooks AT Liu Y Reynolds SL Yanagisawa LL Benveniste EN. SOCS3 deficiency promotes M1 macrophage polarization and inflammation. J Immunol (2012) 189:3439–48. doi: 10.4049/jimmunol.1201168 PMC4184888 22925925

[16] Ohno-Urabe S Aoki H Nishihara M Furusho A Hirakata S Nishida N. Role of macrophage SOCS3 in the pathogenesis of aortic dissection. J Am Heart Assoc (2018) 7:e007389. doi: 10.1161/JAHA.117.007389 29343476 PMC5850160

[17] Liongue C O’Sullivan LA Trengove MC Ward AC. Evolution of JAK-STAT pathway components: mechanisms and role in immune system development. PloS One (2012) 7:e32777. doi: 10.1371/journal.pone.0032777 22412924 PMC3296744

[18] Lawrence C. The husbandry of zebrafish (Danio rerio): a review. Aquaculture (2007) 269:1–20. doi: 10.1016/j.aquaculture.2007.04.077

[19] Dobrzycki T Krecsmarik M Monteiro R. Genotyping and quantification of *in situ* hybridization staining in zebrafish. J Visual Experiments (2020) 155:e59956. doi: 10.3791/59956 32065138

[20] Buchan KD Prajsnar TK Ogryzko NV de Jong NWM van Gent M Kolata J. A transgenic zebrafish line for *in vivo* visualisation of neutrophil myeloperoxidase. PloS One (2019) 14:e0215592. doi: 10.1371/journal.pone.0215592 31002727 PMC6474608

[21] Ellett F Pase L Hayman JW Andrianopoulos A Lieschke GJ. mpeg1 promoter transgenes direct macrophage-lineage expression in zebrafish. Blood (2011) 117:e49–56. doi: 10.1182/blood-2010-10-314120 PMC3056479 21084707

[22] Thisse C Thisse B. High-resolution *in situ* hybridization to whole-mount zebrafish embryos. Nat Protoc (2008) 3:59. doi: 10.1038/nprot.2007.514 18193022

[23] Le Guyader D Redd MJ Colucci-Guyon E Murayama E Kissa K Briolat V. Origins and unconventional behavior of neutrophils in developing zebrafish. Blood (2008) 111:132–41. doi: 10.1182/blood-2007-06-095398 17875807

[24] Schneider CA Rasband WS Eliceiri KW. NIH Image to ImageJ: 25 years of image analysis. Nat Methods (2012) 9:671–5. doi: 10.1038/nmeth.2089 PMC5554542 22930834

[25] Lieschke GJ Oates AC Paw BH Thompson MA Hall NE Ward AC. Zebrafish SPI-1 (PU.1) marks a site of myeloid development independent of primitive erythropoiesis: implications for axial patterning. Dev Biol (2002) 246:274–95. doi: 10.1006/dbio.2002.0657 12051816

[26] Almohaisen FLJ Heidary S Sobah ML Ward AC Liongue C. B cell lymphoma 6A regulates immune development and function in zebrafish. Front Cell Infect Microbiol (2022) 12:887278. doi: 10.3389/fcimb.2022.887278 36389136 PMC9650189

[27] Basheer F Lee E Liongue C Ward AC. Zebrafish model of severe combined immunodeficiency (SCID) due to JAK3 mutation. Biomolecules (2022) 12:1521. doi: 10.3390/biom12101521 36291730 PMC9599616

[28] de Oliveira S Reyes-Aldasoro CC Candel S Renshaw SA Mulero V Calado A. Cxcl8 (IL-8) mediates neutrophil recruitment and behavior in the zebrafish inflammatory response. J Immunol (2013) 190:4349–59. doi: 10.4049/jimmunol.1203266 PMC3736093 23509368

[29] Moore FE Garcia EG Lobbardi R Jain E Tang Q Moore JC. Single-cell transcriptional analysis of normal, aberrant, and malignant hematopoiesis in zebrafish. J Exp Med (2016) 213:979–92. doi: 10.1084/jem.20152013 PMC4886368 27139488

[30] Sertori R Jones R Basheer F Rivera L Dawson S Loke S. Generation and characterization of a zebrafish IL-2Rgc SCID model. Int J Mol Sci (2022) 23:2385. doi: 10.3390/ijms23042385 35216498 PMC8875600

[31] Rao X Huang X Zhou Z Lin X. An improvement of the 2ˆ(-delta delta CT) method for quantitative real-time polymerase chain reaction data analysis. Biostat Bioinf Biomath (2013) 3:71–85. PMC4280562 25558171

[32] Petrie TA Strand NS Yang CT Rabinowitz JS Moon RT. Macrophages modulate adult zebrafish tail fin regeneration. Development (2014) 141:2581–91. doi: 10.1242/dev.098459 PMC4067955 24961798

[33] Liongue C Hall CJ O’Connell BA Crosier P Ward AC. Zebrafish granulocyte colony-stimulating factor receptor signaling promotes myelopoiesis and myeloid cell migration. Blood (2009) 113:2535–46. doi: 10.1182/blood-2008-07-171967 19139076

[34] Resource Coordinators NCBI. Database resources of the National Center for Biotechnology Information. Nucleic Acids Res (2016) 44:D7–19. doi: 10.1093/nar/gkv1290 26615191 PMC4702911

[35] Liu F Wen Z. Cloning and expression pattern of the lysozyme c gene in zebrafish. Mech Dev (2002) 113:69–72. doi: 10.1016/S0925-4773(01)00658-X 11900976

[36] Herbomel P Thisse B Thisse C. Ontogeny and behaviour of early macrophages in the zebrafish embryo. Development (1999) 126:3735–45. doi: 10.1242/dev.126.17.3735 10433904

[37] Zakrzewska A Cui C Stockhammer OW Benard EL Spaink HP Meijer AH. Macrophage-specific gene functions in Spi1-directed innate immunity. Blood (2010) 116:e1–e11. doi: 10.1182/blood-2010-01-262873 20424185

[38] Lieschke GJ Oates AC Crowhurst MO Ward AC Layton JE. Morphologic and functional characterization of granulocytes and macrophages in embryonic and adult zebrafish. Blood (2001) 98:3087–96. doi: 10.1182/blood.V98.10.3087.h8003087_3087_3096 11698295

[39] Brownlie A Hersey C Oates AC Paw BH Falick AM Witkowska HE. Characterization of embryonic globin genes of the zebrafish. Dev Biol (2003) 255:48–61. doi: 10.1016/S0012-1606(02)00041-6 12618133

[40] Willett CE Cherry JJ Steiner LA. Characterization and expression of the recombination activating genes (rag1 and rag2) of zebrafish. Immunogenetics (1997) 45:394–404. doi: 10.1007/s002510050221 9089097

[41] Hasegawa T Hall CJ Crosier PS Abe G Kawakami K Kudo A. Transient inflammatory response mediated by interleukin-1beta is required for proper regeneration in zebrafish fin fold. Elife (2017) 6:22716. doi: 10.7554/eLife.22716 PMC5360449 28229859

[42] Nguyen-Chi M Laplace-Builhe B Travnickova J Luz-Crawford P Tejedor G Phan QT. Identification of polarized macrophage subsets in zebrafish. Elife (2015) 4:e07288–8. doi: 10.7554/eLife.07288 PMC4521581 26154973

[43] Rasighaemi P Basheer F Liongue C Ward AC. Zebrafish as a model for leukemia and other hematopoietic disorders. J Hematol Oncol (2015) 8:29. doi: 10.1186/s13045-015-0126-4 25884214 PMC4389495

[44] Miao KZ Kim GY Meara GK Qin X Feng H. Tipping the scales with zebrafish to understand adaptive tumor immunity. Front Cell Dev Biol (2021) 9:660969. doi: 10.3389/fcell.2021.660969 34095125 PMC8173129

[45] Liu X Li Y-S Shinton SA Rhodes J Tang L Feng H. Zebrafish B cell development without a pre–B cell stage, revealed by CD79 fluorescence reporter transgenes. J Immunol (2017) 199:1706–15. doi: 10.4049/jimmunol.1700552 PMC5563169 28739882

[46] Pereiro P Varela M Diaz-Rosales P Romero A Dios S Figueras A. Zebrafish NK lysins: First insights about their cellular and functional diversification. Dev Comp Immunol (2015) 51:148–59. doi: 10.1016/j.dci.2015.03.009 25813149

[47] Oltova J Svoboda O Bartunek P. Hematopoietic cytokine gene duplication in zebrafish erythroid and myeloid lineages. Front Cell Dev Biol (2018) 6:174. doi: 10.3389/fcell.2018.00174 30619854 PMC6306437

[48] Liao EC Paw BH Oates AC Pratt SJ Postlethwait JH Zon LI. SCL/Tal-1 transcription factor acts downstream of cloche to specify hematopoietic and vascular progenitors in zebrafish. Genes Dev (1998) 12:621–6. doi: 10.1101/gad.12.5.621 PMC316577 9499398

[49] Hansen JD Zapata AG. Lymphocyte development in fish and amphibians. Immunol Rev (1998) 166:199–220. doi: 10.1111/j.1600-065X.1998.tb01264.x 9914914

[50] Bertrand JY Kim AD Violette EP Stachura DL Cisson JL Traver D. Definitive hematopoiesis initiates through a committed erythromyeloid progenitor in the zebrafish embryo. Development (2007) 134:4147–56. doi: 10.1242/dev.012385 PMC2735398 17959717

[51] Stujenske JM Dowling JE Emran F. The bugeye mutant zebrafish exhibits visual deficits that arise with the onset of an enlarged eye phenotype. Invest Ophthalmol Visual Sci (2011) 52:4200–7. doi: 10.1167/iovs.10-6434 PMC3175981 21460263

[52] Miyazawa R Matsuura Y Shibasaki Y Imamura S Nakanishi T. Cross-reactivity of monoclonal antibodies against CD4-1 and CD8α of ginbuna crucian carp with lymphocytes of zebrafish and other cyprinid species. Dev Comp Immunol (2018) 80:15–23. doi: 10.1016/j.dci.2016.12.002 27965016

[53] Zimmerman AM Moustafa FM Romanowski KE Steiner LA. Zebrafish immunoglobulin IgD: unusual exon usage and quantitative expression profiles with IgM and IgZ/T heavy chain isotypes. Mol Immunol (2011) 48:2220–3. doi: 10.1016/j.molimm.2011.06.441 PMC3163713 21820179

[54] Bottiglione F Dee CT Lea R Zeef LAH Badrock AP Wane M. Zebrafish IL-4-like cytokines and IL-10 suppress fnflammation but only IL-10 is essential for gill homeostasis. J Immunol (2020) 205:994–1008. doi: 10.4049/jimmunol.2000372 32641385 PMC7416321

[55] Aggad D Stein C Sieger D Mazel M Boudinot P Herbomel P. *In vivo* analysis of ifn-γ1 and ifn-γ2 signaling in zebrafish. J Immunol (2010) 185:6774–82. doi: 10.4049/jimmunol.1000549 21048110

[56] Croker BA Krebs DL Zhang J-G Wormald S Willson TA Stanley EG. SOCS3 negatively regulates IL-6 signaling in vivo. Nat Immunol (2003) 4:540. doi: 10.1038/ni931 12754505

[57] Wong PK Egan PJ Croker BA O’Donnell K Sims NA Drake S. SOCS-3 negatively regulates innate and adaptive immune mechanisms in acute IL-1-dependent inflammatory arthritis. J Clin Invest (2006) 116:1571–81. doi: 10.1172/JCI25660 PMC1462939 16710471

[58] Xiong S Wu J Jing J Huang P Li Z Mei J. Loss of stat3 function leads to spine malformation and immune disorder in zebrafish. Sci Bull (2017) 62:185–96. doi: 10.1016/j.scib.2017.01.008 36659403

[59] Roberts AW Robb L Rakar S Hartley L Cluse L Nicola NA. Placental defects and embryonic lethality in mice lacking suppressor of cytokine signaling 3. Proc Natl Acad Sci USA (2001) 98:9324–9. doi: 10.1073/pnas.161271798 PMC55419 11481489

[60] Banks KM Lan Y Evans T. Tet proteins regulate neutrophil granulation in zebrafish through demethylation of socs3b mRNA. Cell Rep (2021) 34:108632. doi: 10.1016/j.celrep.2020.108632 33440144 PMC7837371

[61] Basheer F Rasighaemi P Liongue C Ward AC. Zebrafish granulocyte colony-stimulating factor receptor maintains neutrophil number and function throughout the life span. Infect Immun (2019) 87:e00793–18. doi: 10.1128/IAI.00793-18 PMC6346135 30455199

[62] Meier AB Basheer F Sertori R Laird M Liongue C Ward AC. Granulocyte colony-stimulating factor mediated regulation of early myeloid cells in zebrafish. Front Bioscience-Landmark (2022) 27:110. doi: 10.31083/j.fbl2704110 35468669

[63] Boniakowski AE Kimball AS Joshi A Schaller M Davis FM denDekker A. Murine macrophage chemokine receptor CCR2 plays a crucial role in macrophage recruitment and regulated inflammation in wound healing. Eur J Immunol (2018) 48:1445–55. doi: 10.1002/eji.201747400 PMC6371802 29879295

[64] Willenborg S Lucas T van Loo G Knipper JA Krieg T Haase I. CCR2 recruits an inflammatory macrophage subpopulation critical for angiogenesis in tissue repair. Blood (2012) 120:613–25. doi: 10.1182/blood-2012-01-403386 22577176

[65] Torraca V Cui C Boland R Bebelman J-P van der Sar AM Smit MJ. The CXCR3-CXCL11 signaling axis mediates macrophage recruitment and dissemination of mycobacterial infection. Dis Models Mech (2015) 8:253–69. doi: 10.1242/dmm.017756 PMC4348563 25573892

[66] Brender C Tannahill GM Jenkins BJ Fletcher J Columbus R Saris CJ. Suppressor of cytokine signaling 3 regulates CD8 T-cell proliferation by inhibition of interleukins 6 and 27. Blood (2007) 110:2528–36. doi: 10.1182/blood-2006-08-041541 17609432

[67] Schwartzman S. Advancements in the management of uveitis. Best Pract Res Clin Rheumatol (2016) 30:304–15. doi: 10.1016/j.berh.2016.07.005 27886802

[68] Yu C-R Kim S-H Mahdi RM Egwuagu CE. SOCS3 deletion in T lymphocytes suppresses development of chronic ocular inflammation *via* upregulation of CTLA-4 and expansion of regulatory T cells. J Immunol (2013) 191:5036–43. doi: 10.4049/jimmunol.1301132 PMC4041279 24101549

[69] Chen M Zhao J Ali IHA Marry S Augustine J Bhuckory M. Cytokine signaling protein 3 deficiency in myeloid cells promotes retinal degeneration and angiogenesis through arginase-1 up-regulation in experimental autoimmune uveoretinitis. Am J Pathol (2018) 188:1007–20. doi: 10.1016/j.ajpath.2017.12.021 29452101

[70] Yan Z Yang W Parkitny L Gibson SA Lee KS Collins F. Deficiency of Socs3 leads to brain-targeted EAE *via* enhanced neutrophil activation and ROS production. JCI Insight (2019) 5:e126520. doi: 10.1172/jci.insight.126520 30939124 PMC6538334

[71] Veth KN Willer JR Collery RF Gray MP Willer GB Wagner DS. Mutations in zebrafish lrp2 result in adult-onset ocular pathogenesis that models myopia and other risk factors for glaucoma. PloS Genet (2011) 7:e1001310. doi: 10.1371/journal.pgen.1001310 21379331 PMC3040661

[72] Ozawa Y Nakao K Kurihara T Shimazaki T Shimmura S Ishida S. Roles of STAT3/SOCS3 pathway in regulating the visual function and ubiquitin-proteasome-dependent degradation of rhodopsin during retinal inflammation. J Biol Chem (2008) 283:24561–70. doi: 10.1074/jbc.M802238200 PMC2528996 18614536

[73] Carow B Rottenberg ME. SOCS3, a major regulator of infection and inflammation. Front Immunol (2014) 5:58. doi: 10.3389/fimmu.2014.00058 24600449 PMC3928676

[74] White GE Cotterill A Addley MR Soilleux EJ Greaves DR. Suppressor of cytokine signalling protein SOCS3 expression is increased at sites of acute and chronic inflammation. J Mol Histol (2011) 42:137–51. doi: 10.1007/s10735-011-9317-7 PMC3070874 21360047

[75] Hadjadj J Castro CN Tusseau M Stolzenberg MC Mazerolles F Aladjidi N. Early-onset autoimmunity associated with SOCS1 haploinsufficiency. Nat Commun (2020) 11:5341. doi: 10.1038/s41467-020-18925-4 33087723 PMC7578789

[76] Yang XO Zhang H Kim B-S Niu X Peng J Chen Y. The signaling suppressor CIS controls proallergic T cell development and allergic airway inflammation. Nat Immunol (2013) 14:732–40. doi: 10.1038/ni.2633 PMC4084713 23727894

[77] Louis C Guimaraes F Yang Y D’silva D Kratina T Dagley L. NK cell–derived GM-CSF potentiates inflammatory arthritis and is negatively regulated by CIS. J Exp Med (2020) 217:e20191421. doi: 10.1084/jem.20191421 32097462 PMC7201918

[78] Takase H Yu CR Liu X Fujimoto C Gery I Egwuagu CE. Induction of suppressors of cytokine signaling (SOCS) in the retina during experimental autoimmune uveitis (EAU): potential neuroprotective role of SOCS proteins. J Neuroimmunol (2005) 168:118–27. doi: 10.1016/j.jneuroim.2005.07.021 16154209

[79] Ahmed CM Massengill MT Brown EE Ildefonso CJ Johnson HM Lewin AS. A cell penetrating peptide from SOCS-1 prevents ocular damage in experimental autoimmune uveitis. Exp Eye Res (2018) 177:12–22. doi: 10.1016/j.exer.2018.07.020 30048621 PMC6528831

[80] He C Yu C-R Mattapallil MJ Sun L Larkin J III Egwuagu CE. SOCS1 mimetic peptide suppresses chronic intraocular inflammatory disease (uveitis). Med Inflamm (2016) 2016:2939370. doi: 10.1155/2016/2939370 PMC5040805 27703302

[81] Jo D Liu D Yao S Collins RD Hawiger J. Intracellular protein therapy with SOCS3 inhibits inflammation and apoptosis. Nat Med (2005) 11:892–8. doi: 10.1038/nm1269 16007096

[82] Fletcher TC DiGiandomenico A Hawiger J. Extended anti-inflammatory action of a degradation-resistant mutant of cell-penetrating suppressor of cytokine signaling 3. J Biol Chem (2010) 285:18727–36. doi: 10.1074/jbc.M109.095216 PMC2881796 20400504

