# Supplementary material for: Socs3b regulates the development and function of innate immune cells in zebrafish
Source: Front Immunol. 2023 Mar 8;14:1119727. doi: 10.3389/fimmu.2023.1119727 (PMC10030509; doi:10.3389/fimmu.2023.1119727)
Supplement: Supplementary file 1 [file DataSheet_1.pdf]

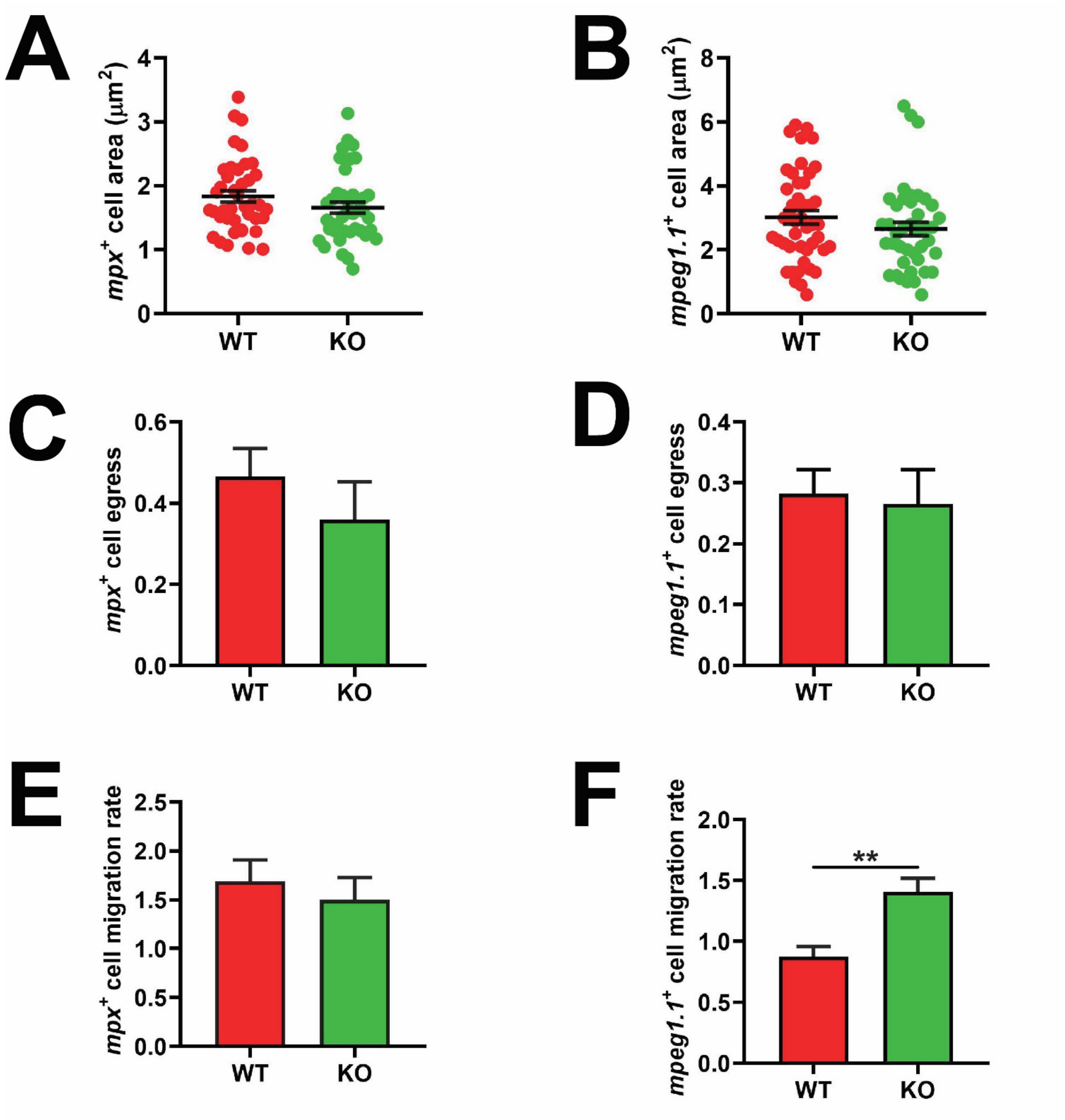

Supplementary Figure 1. Effect of *socs3b* ablation on innate immune cells following wounding. Quantitation of size of *mpx*$^{+}$ (A) and *mpeg1.1*$^{+}$ (B) cells present at the site of injury during peak cell migration, rate of egress of *mpx*$^{+}$ (C) and *mpeg1.1*$^{+}$ (D) cells and cell migration rate of of *mpx*$^{+}$ (E) and *mpeg1.1*$^{+}$ (F) cells in WT and KO embryos, with mean and SEM indicated. (n = 8-12) (*** $p <$ 0.001** $p < 0.01$, * $p < 0.05$)

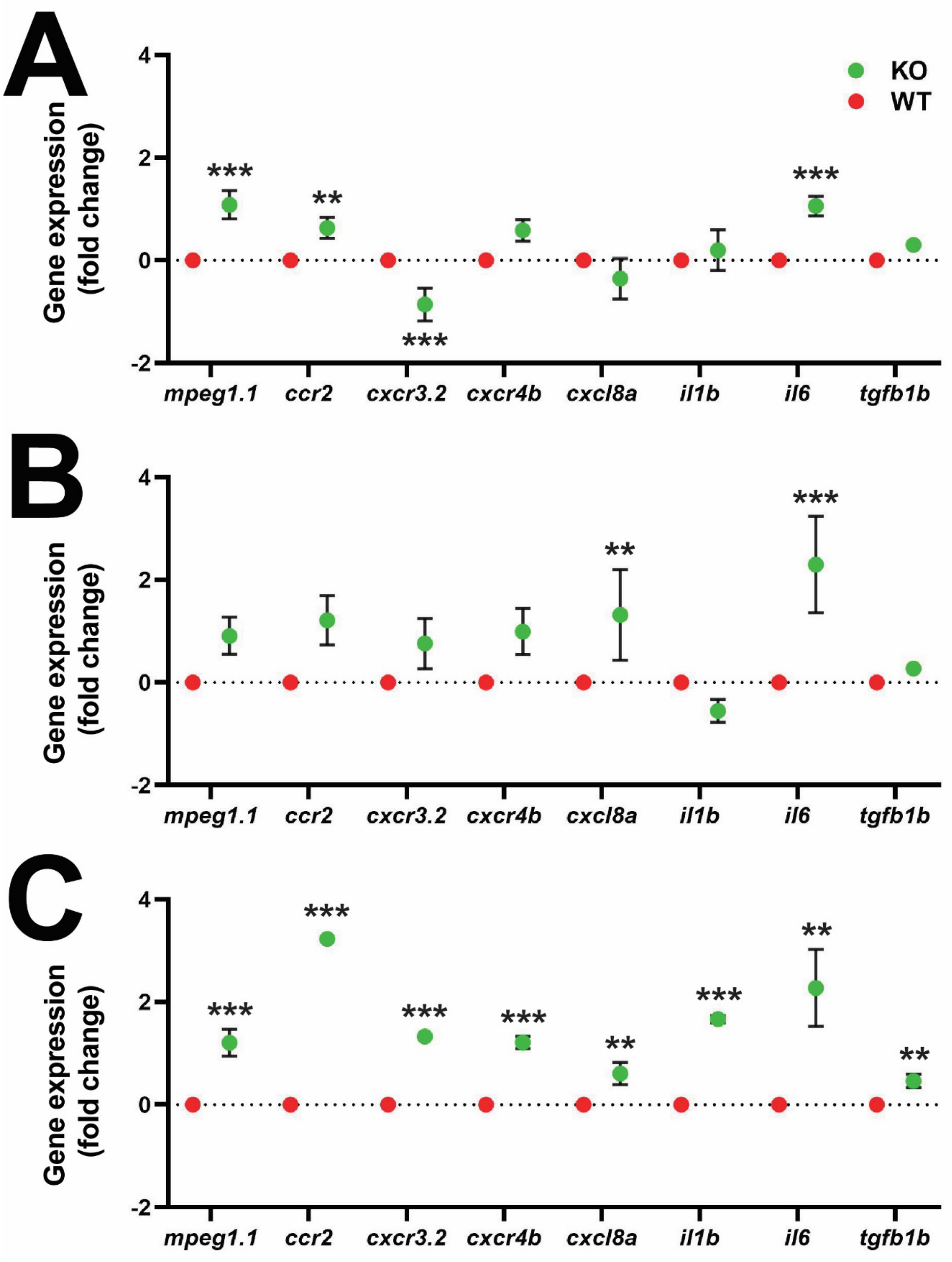

Supplementary Figure 2. Effect of *socs3b* ablation on expression of inflammatory genes following wounding. Gene expression analysis of the indicated genes in WT and KO embryos subjected to wounding presented as fold-change (log_2) relative to WT at 0 hpw (A), 4 hpw (B) and 8 hpw (C), showing mean and SEM (n = 8-12).

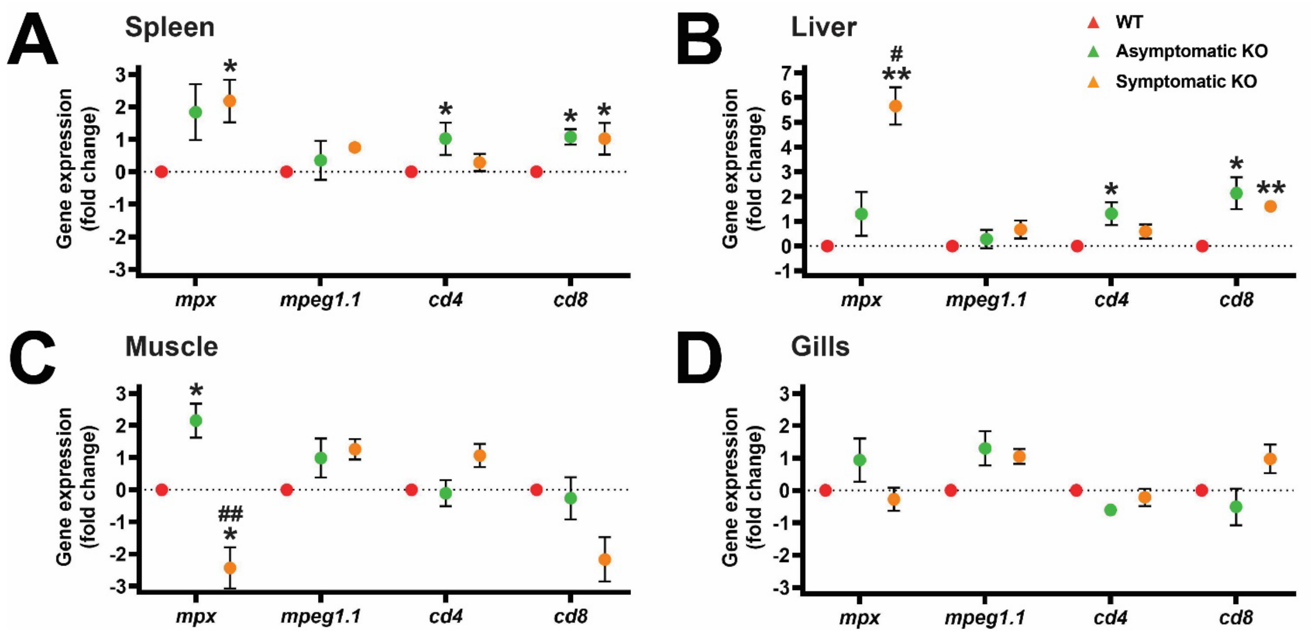

Supplementary Figure 3. Expression of key immune genes in select tissues. Relative expression of the indicated myeloid and lymphoid marker genes in spleen (A), liver (B), muscle (C) and gills (D) in WT and KO fish presented as fold change (log_2) compared to WT, showing mean and SEM, with statistical significance indicated (relative to WT: ** $p < 0.01$, * $p < 0.05$; relative to KO: $^{\#\#}$ $p<0.01$, $^{\#}$ $p<0.05$, n=6).

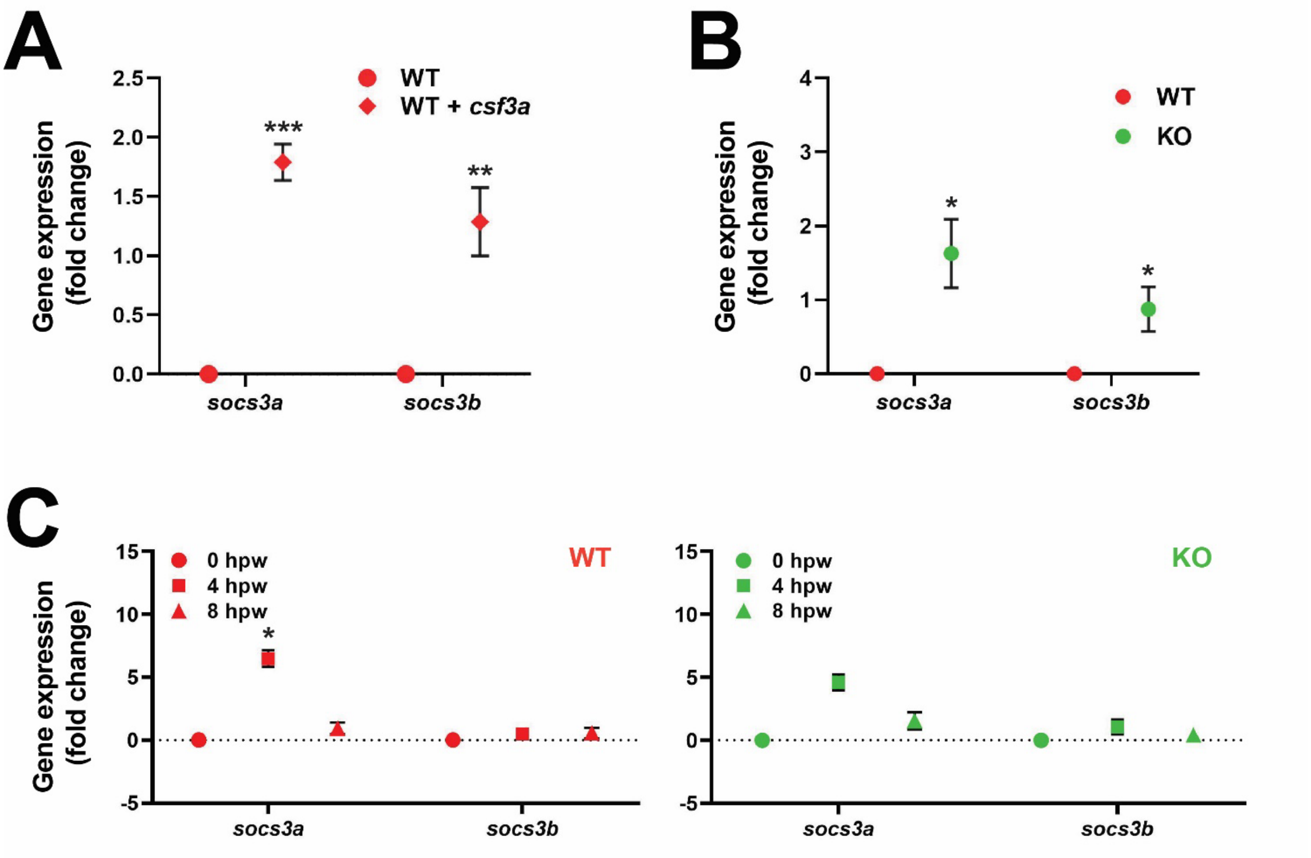

Supplementary Figure 4. Expression analysis of *socs3* paralogues. Gene expression analysis of *socs3a* and *socs3b* in uninjected WT embryos or those injected with *csf3a* mRNA (+ *csf3*) injections presented as fold-change (log_2) relative to WT uninjected embryos, showing mean and SEM, with statistical significance indicated (n = 3) (A). Gene expression analysis of *socs3a* and *socs3b* in WT and KO embryos at 5 dpf presented as fold-change (log_2) relative to WT, showing mean and SEM (n = 4) (B). Gene expression analysis of *socs3a* and *socs3b* in WT and KO embryos subjected to wounding presented as fold-change (log_2) relative to 0 hpw showing mean and SEM (C) (*** $p < 0.001$, ** $p < 0.01$, * $p < 0.05$).
